# A new method for disease diagnosis based on hierarchical BRB with power set

**DOI:** 10.1016/j.heliyon.2023.e13619

**Published:** 2023-02-11

**Authors:** Wence Han, Xiao Kang, Wei He, Li Jiang, Hongyu Li, Bing Xu

**Affiliations:** a Harbin Normal University, Harbin 150025, China; b Rocket Force University of Engineering, Xi'an 710025, China; c Harbin Medical University Cancer Hospital, China

**Keywords:** Disease diagnosis, Belief rule base, Power set, Local ignorance

## Abstract

Disease diagnosis occupies an important position in the medical field. The diagnosis of the disease is the basis for choosing the right treatment plan. Doctors must first diagnose what the patient has based on the clinical characteristics of various diseases, and then they can administer the right medicine. When building models for disease diagnosis, models are required to be able to handle various uncertainty information. The belief rule base (BRB) can effectively handle various information under uncertainty by introducing belief distributions. However, in current research, BRB-based disease diagnosis models still have problems of combinatorial rule explosion and inability to deal with local ignorance effectively. Therefore, a hierarchical BRB with power set (H-BRBp)-based disease diagnosis model is proposed in this paper. First, the physiological indexes and data of the patients were analyzed, and the data were preprocessed using the principal component regression (PCR) algorithm. Second, the H-BRBp disease diagnosis model was constructed to solve the deficiencies in the above BRB disease diagnosis model. Finally, the validity and advantages of the model were verified by experiments on lumbar spine disease diagnosis and a large number of comparison experiments.

## 1 Introduction

Disease diagnosis is a process by which a doctor determines whether a patient has a disease based on data such as the patient's disease symptoms, adverse reactions, and test results. And to determine the type of disease the patient has [1]. In an actual disease diagnosis environment. Especially when there is an overwhelming number of patients and the amount of data to be processed is too large, it may be difficult for doctors to handle in a short period of time. Therefore, in recent years, researchers have been working on applying algorithms and techniques of artificial intelligence (AI) to disease diagnosis. Using algorithms and technologies from the field of AI to have been used build a disease diagnosis model with high reliability and accuracy.

In the existing AI domain model construction, models are generally classified into three categories according to their structure and operation mechanism. Namely, data-driven approach, model-analytic approach and hybrid information model approach. This classification method has been widely used in the field of industrial fault diagnosis [2]. In this paper, based on the original classification combined with the characteristics of disease diagnosis model data types and model operation mechanism, the disease diagnosis models are summarized and classified into the following three types: based on quantitative information, qualitative knowledge and semi-quantitative information. Diagnosis models for diseases based on quantitative information are constructed using data-driven methods from observed data. For example, Hu, F et al. used BP neural network (BP) algorithm to diagnose and predict coronary heart disease and breast cancer and improved the convergence speed and numerical stability of the model [3]. Asadi, S et al. constructed a new cardiac disease diagnosis model using the random forest (RF) algorithm. A certain number of diversified trees are generated and they make an accurate decision together. Simultaneously, the number of trees is determined [4]. Li X et al. designed a regional aggregation GCN (RAGCN). Key regions of hand bones were defined according to clinical criteria, and independent CNN paths were established to extract features from different key regions to achieve the assessment of bone age. The model can aggregate the regional features into the overall bone age representation based on the regional adjacency. RAGCN can also infer the importance of different regions in the feature aggregation process [5]. Li X et al. designed a lesion attention pyramid network (LAPN). The model integrates sub-networks with different resolutions to obtain multi-scale features. A lesion attention module (LAM) was used to capture the complementary relationship between high-resolution features and low-resolution features and to fuse lesion activation maps. The model can provide lesion activation maps with lesion consistency as additional evidence for clinical diagnosis [6]. Alfaer N M et al. designed an automated brain hemorrhage diagnosis using a fusion-based deep learning and population intelligence (AICH-FDLSI) algorithm. The model has four main stages, preprocessing, image segmentation, feature extraction and classification. In addition, the model uses the Deer Hunting Optimization (DHO) algorithm for hyperparameter optimization of the CapsNet and DenseNet models. The effectiveness and superiority of the model are also verified experimentally [7]. Zhao, H et al. applied the K-nearest neighbors (KNN) algorithm to the diagnosis of Parkinson's disease. Gait features, such as time and force features, as well as their coefficient of variance and asymmetry index, were extracted and compared. Overcoming imbalances during severity levels affects diagnosis [8]. Bi, X et al. discussed the classification problem for the detection of Alzheimer's disease (AD) and proposed an AD disease diagnosis model with the enhancement structure of an extreme learning machine (ELM). Through experiments, it was verified that this method has good learning ability in AD disease diagnosis [9]. The disease diagnosis model based on quantitative information does not need complicated mechanism analysis, but the output results are very dependent on data samples, and sometimes the output results even violate basic medical knowledge.

Different from abovementioned methods, the disease diagnosis model based on qualitative knowledge is constructed according to the medical mechanism. For example, Moraes Lo et al. used the decision tree method to make differential diagnosis of distinct WHO categories of B-cell chronic lymphoproliferative disorders using flow cytometry data. The whole process is performed through seven binary transparent decision nodes [10]. Urrea, C et al. constructed a new expert system using relevant risk factors and three algorithms for diagnosis criteria based on WHO criteria for clinical variables. Pre-diagnosis of hypertension, diabetes mellitus type 2 and metabolic syndrome was achieved [11]. Li, J et al. improved the D-S evidence theory (DS) based on multimodal cardiac function signals. The classification accuracy of the algorithm was improved. This algorithm was used as the basis for the actual construction of the heart disease diagnosis model [12]. Lo, C et al. used the analytic hierarchy process (AHP) to determine critical variables influencing delayed diagnosis. These variables were used to construct models for predicting delayed diagnosis in patients with hematuria through multiple machine learning techniques. This model was successfully applied to the diagnosis of bladder cancer [13]. A disease diagnosis model based on qualitative knowledge that does not depend on observational data. However, due to the complex internal structure of the model, there are often many interfering factors in the diagnosis of diseases in reality, and the accuracy of the model is usually limited.

The semi-quantitative information-based disease diagnosis method is a model that lies between quantitative information and qualitative knowledge. It can effectively integrate quantitative information and qualitative knowledge. For example, Bamunu Mudiyanselage, T et al. used fuzzy neural networks to address uncertainty in genetic data. A new hybrid algorithm for data preprocessing was proposed to achieve accurate cancer detection [14]. Balasubramanian, K et al. improved firefly swarm and differential optimization algorithms by using adaptive fuzzy neural inference. A disease diagnosis model was proposed for the detection of diseases such as breast cancer and glaucoma [15]. Ahmed, F et al. accurately and rapidly identified critically ill and ill COVID-19 patients using a belief rule base (BRB) model. The risk of hospital overload was reduced. It provides an effective reference for treatment plan selection for COVID-19 patients [16]. Kong, G et al. used BRB for cardiac pain diagnosis. The risk classification of patients with cardiac chest pain was successfully achieved by adjusting BRB with accumulated historical clinical cases [17]. Compared with quantitative information models semi-quantitative information models can ensure model accuracy while reducing the dependence of model output results on data samples. Compared with qualitative knowledge models, semi-quantitative information models reduce the complexity of the internal structure of their models, strengthen the resistance of the models to interference, and enable the models to better handle various uncertain information.

BRB is a typical semi-quantitative information-based model proposed by Yang et al. [18]. Its modeling process is to construct the model by expert knowledge. The model is trained by using data samples. The expert knowledge is effectively integrated in the process of expression and inference. BRB has the ability is able to handle various uncertainty information. It also has better stability and model interpretability [19] compared to data-driven models. Because BRB uses labeled data for modeling, there is no need to use enhancement techniques. BRB has been widely used in fault detection [20], safety assessment [21], aerospace [22], medical [23] and other fields. At present, BRB has been proved that it can perform well in the field of disease diagnosis. However, in the actual disease diagnosis environment, there are still problems of rule explosion caused by too many input attributes and local ignorance due to the similarity of attributes between different output results.

Therefore, the motivation of this paper is to solve the problem of combinatorial rule explosion caused by excessive input attributes of BRB in the disease diagnosis domain. And the local ignorance problem caused by the highly similar characteristic attributes of different disease types in the actual disease diagnosis context. Taking the diagnosis of lumbar spine diseases as an example, the lumbar spine is an important part of the medial skeleton and consists of many parts. These parts include intervertebral discs, nerves, muscles, medulla and a group of vertebrae. The main functions of the lumbar spine are to support the body, protect the spinal cord medulla and nerve centers, and facilitate body movement. Several causes related to life conditions and habits can lead to different lumbar spine disorders. Herniated discs and spondylolisthesis are the main disorders associated with the lumbar spine. In the case of spinal slippage, a doctor can diagnose it by examining the spinal slippage level. However, for herniated discs, patients with herniated discs often have examination findings that are very similar to those of a healthy lumbar spine (e.g., pelvic incidence, pelvic tilt, lumbar lordosis angle, and pelvic radius). Therefore, for disease diagnosis modeling, the output has a local ignorance problem between the normal lumbar spine and disc herniation. The power set BRB can solve this problem by assigning a confidence level to the intervals where local ignorance information exists.

In addition, the number of rules in BRB is based on the Cartesian product algorithm. Therefore, when there are too many input attributes, there will be an explosion of combined rules, which leads to an overly complex model and often reduces the accuracy of the model. In the experimental session of this paper, for example, we selected four attributes, namely, spinal slippage classification, lumbar anterior convexity angle, pelvic tilt, and sacral slope, as input, and then the experts gave four reference values for each attribute. Then, the number of rules for BRB is 256. However, for hierarchical BRB (HBRB), the expert only needs to determine the hierarchical structure of HBRB by mechanistic analysis. So, the number of rules in each sub rule base of HBRB is controlled in a small range. Then, the overall number of rules of the model will be greatly reduced. For example, the four attributes are divided into three levels. Each layer has two attributes, and each attribute has four reference values. Then, the number of rules in each layer is 16, and the number of rules in the hierarchical HBRB model is 48. Compared with BRB, the number of rules of HBRB is reduced by 81.25%.

In summary, to solve the above problems. In this paper, a hierarchical BRB with a power set disease diagnosis model is proposed. The main contributions of this paper are as follows.

1) Compared with previous work, this paper presents the H-BRBp disease diagnosis model. This model uses the power set approach for the first time to solve the local ignorance problem caused by similar input attributes in the disease diagnosis model. Based on this, this paper uses a hierarchical approach to solve the problem of combinatorial rule explosion in the BRB disease diagnosis model.
2) In this paper, effective data preprocessing is performed before the construction of disease diagnosis model for the irrelevant and interfering attributes that exist in the actual disease diagnosis environment. The complexity of model construction is reduced, and the interference resistance of the disease diagnosis model is enhanced.
3) The proposed model can be used to disease diagnosis in different medical fields according to different medical specialists. Therefore, the model is highly adaptable and easy to extend. Additionally, because expert knowledge is cited as hyperparameters, its model results are constrained by expert knowledge. Therefore, the model output results are more consistent with basic common sense, while making the model somewhat interpretable and retrievable.

The remainder of the paper is structured as follows. In Section 2, common problems in disease diagnosis are described, and the problem is formulated. In Section 3, the H-BRBp disease diagnosis model is constructed, defining the inference and optimization process of the model. In Section 4, the validity and advantages of the model in disease diagnosis are validated using publicly available vertebrate datasets from the UCI machine learning knowledge base. In Section 5, a summary of the paper is presented, and an outlook on future work is provided.

## 2 Problem formulation

When constructing a disease diagnosis model, the following three issues need to be considered:

Problem 1 How to select reasonable input attributes while ensuring the accuracy of the disease type output from the disease diagnosis model and the interpretability of the combination of input attributes. By analyzing the mechanism of disease diagnosis, the selection process of the initial input attributes can be represented by Equation (1).

(1) $$\left\{x_{1},x_{2},\cdots ,x_{M}\right\}=f\left(x_{1},x_{2},\cdots ,x_{Q},\alpha \right)\;\left(M<Q\right)$$

where $\left\{x_{1},x_{2},\cdots ,x_{M}\right\}$ represents the selected attribute set. M represents the number of selected attributes. f(·) represents the selection function. $\left\{x_{1},x_{2},\cdots ,x_{Q}\right\}$ represents the initial attribute set. Q represents the initial attribute number. α represents the mechanistic significance of the properties during the selection process.

Problem 2 How to construct the inference process of the model with the premise of ensuring the rationality and accuracy of the disease diagnosis model is crucial for the disease diagnosis results. The inference process of the disease diagnosis model can be represented by Equation (2).

(2) $$y=g\left(x_{1},x_{2},\cdots ,x_{M},\Omega \right)$$

where Ω represents the parameter set in the model inference process. y represents the output of the model. g(·) represents the inference function of the model.

Problem 3 How to reasonably optimize the initial output results of disease diagnosis models is essential for the accuracy of the output results of disease diagnosis models. Model optimization largely affects the output results of disease diagnosis models. The optimization process of the model can be represented by Equation (3).

(3) $$\Omega =h\left(y,x_{1},\cdots ,x_{M},\chi \right)$$

where h(·) represents the optimization function. χ represents the set of parameters in the optimization process.

## 3 H-BRBp disease diagnosis model

By analyzing the above issues, this section defines the modeling process of the H-BRBp disease diagnosis model. Section 3.1 defines the basic structure of the model. Section 3.2 uses principal component regression (PCR) to select the attributes that have a high impact on the disease diagnosis model. Section 3.3 defines the inference process of the model. Section 3.4 defines the optimization process of the model. Section 3.5 summarizes sections 1, 2, 3.4 and defines the thought process for application development of the H-BRBp disease diagnosis model.

### 3.1 Definition of the basic structure of the H-BRBp disease diagnosis model

The rules of BRB are usually determined by experts in the field based on empirical knowledge with historical data of the system model. In BRB, there are many if-then rules. They use the belief degree to represent various types of uncertain information, including ambiguity, randomness, and ignorance [24]. The expert sets the initial values of the important parameters by a number of rules with confidence distributions. The expert knowledge is embedded into the rules to form a belief rule base [25].

On the basis of BRB. The hierarchical structure BRB model is composed of several sub-rule bases. Each sub rule base consists of a certain number of rules. It can well solve the problem of rule explosion caused by too many input attributes [26]. The rules in HBRB are represented by Equation (4).

(4) $$\begin{array}{l} BRB_{u}^{g}\text{:}\\ If\;x_{1}{\text{is}\;A}_{1}^{k}\wedge x_{2}{\text{is}\;A}_{2}^{k}\wedge \cdots \wedge x_{M}{\text{is}\;A}_{M}^{k}\\ Then\;y_{k}^{g}\;\text{is}\;\left\{\left(D_{1},\beta_{1}\right),\left(D_{2},\beta_{2}\right),\cdots ,\left(D_{N},\beta_{N}\right)\right\}\\ \text{with}\;\text{rule}\;\text{weight}\;\theta_{k}\\ and\;\text{attribute}\;\text{weight}\;\delta_{1},\delta_{2},\cdots ,\delta_{M} \end{array}$$

where $BRB_{j}^{i}$ represents the jth BRB rule base in the ith layer. $x_{i}$ represents the input attribute. $A_{i}^{k}\left(i=1,2,\cdots M\right)$ represents the reference value of the attribute input. $D_{i}$ represents the type of the output result. $\beta_{i}\left(i=1,2,\cdots ,N\right)$ represents the belief degree of the corresponding reference level. $\theta_{k}$ represents the rule weights, and $\delta_{M}$ represents the attribute weights. $y_{j}^{i}$ represents the output result of the jth BRB rule base in the ith layer. It should be emphasized that in the hierarchical structure, the output result of the ith layer is used as the input of the corresponding BRB rule base in the (i+1)th layer according to the hierarchy classified by the experts. This process is iterated until the final output results are obtained.

HBRB solves the combinatorial rule explosion problem. However, the strong similarity between the parameters of different disease types leads to difficulty in distinguishing specific disease types in the HBRB model. It cannot handle local ignorance information. This reduces the accuracy of the model [27]. To solve this problem, the model is made capable of handling both local ignorance and global ignorance information. Based on the HBRB model, the H-BRBp model is defined using the power set approach. The model definition process is described in detail as follows.

First, the output of the model is defined. In the disease diagnosis model, the disease type is defined as the output of the model, which can be represented by Equation (5).

(5) $$E=\left\{D_{1},D_{2},\cdots ,D_{N}\right\}$$

where D represents the set of disease types. $D_{i}$ represents the ith disease type of the disease diagnosis model species. N represents the number of disease types of the model. In the disease diagnosis model, local ignorance indicates that the disease type may be any J of the N diseases, where J<N. Global ignorance represents the case where the disease type may be any one of all N disease types. The set of disease types with local ignorance and global ignorance can be represented by Equation (6).

(6) $$2^{N}=\left\{\emptyset ,D_{1},D_{2},\cdots ,D_{N},\left\{D_{1},D_{2}\right\},\left\{D_{1},D_{N}\right\},\cdots ,\left\{D_{1},\cdots ,D_{N-1}\right\},℧\right\}$$

where ∅ represents that the disease type of this patient is not in the set of defined disease types. $D_{i}\left(i=1,2,\cdots ,N\right)$ represents that this patient's disease type can be identified as $D_{i}$. $\left\{D_{i},D_{j}\right\}\;\left(i,j=1,2,\cdots ,N\;i\neq j\right)$ represents that this patient's disease type may be either $D_{i}$ or $D_{j}$. ℧ represents that this patient's disease type may be any one of the defined sets of disease types.

**Fig. 1:**
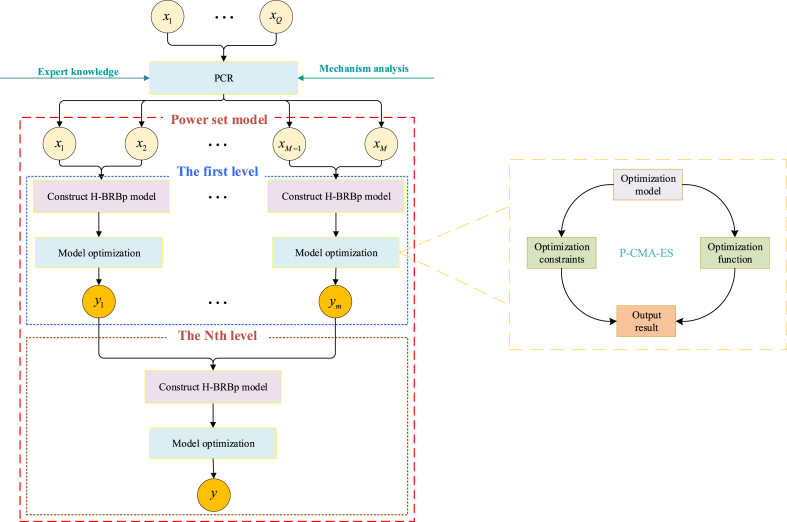
Structure of the H-BRBp disease diagnosis model.

Finally, according to the power set approach defined by Equation (5) and Equation (6). All the sub rule bases in the HBRB rule base shown in Equation (4) are improved to the H-BRBp rule. The overall structure of the H-BRBp disease diagnosis model is shown in Fig. 1. The rules in a certain sub rule base of H-BRBp can be represented by Equation (7).

(7) $$\begin{array}{l} R_{e}\text{:}\\ If\;x_{1}{\text{is}\;A}_{1}^{k}\wedge x_{2}{\text{is}\;A}_{2}^{k}\wedge \cdots \wedge x_{M}{\text{is}\;A}_{M}^{k}\\ Theny_{i}^{j}\;\text{is}\;\left\{\left(D_{1},\beta_{1}\right),\cdots ,\left(D_{N},\beta_{N}\right),\cdots ,\left(\left\{D_{1},\cdots D_{N-1}\right\},\beta_{2^{N-1}}\right),\left(℧,\beta_{2^{N}}\right)\right\}\\ \text{with}\;\text{rule}\;\text{weight}\;\theta_{k}\\ and\;\text{attribute}\;\text{weight}\;\delta_{1},\delta_{2},\cdots ,\delta_{M} \end{array}$$

Where $R_{k}$ represents the kth rule. $\left\{\left(D_{i},D_{j}\right),\beta_{n}\right\}\;\left(i,j=1,2,\cdots ,N\;i\neq j\right)$ represents the degree of belief that the output result is $D_{i}$ or $D_{j}$ is $\beta_{n}$.

### 3.2 Data preprocessing for the PCR-based disease diagnosis model

As there are more attributes affecting disease diagnosis, it increases the workload of data collection. There are often many confounding attributes in actual disease diagnosis. If these attributes are used as the input of the disease diagnosis model, the accuracy of the model will be affected. Therefore, selecting the attributes that have a high impact on disease diagnosis is crucial for the accuracy of the ensuing disease diagnosis model.

There are sometimes correlations between the attributes of different disease types. This increases the complexity of the problem analysis. If each attribute is analyzed separately, the analysis tends to be isolated rather than integrated. Blindly reducing the attributes can lose much information and tend to produce incorrect conclusions. PCR is based on PCA. PCR is able to select several integrated genera among the attributes that are not related to each other. These integrated genera can reflect most of the information provided by all the original attributes. It is also able to eliminate the problem of multicollinearity between variables [28,29]. Therefore, in this paper, PCR is used as a method for selecting the diagnostic attributes of diseases. The main steps of the PCR algorithm are as follows:

Step 1 Centering all samples, which can be expressed by equation (8).

(8) $$x_{i}=x_{i}-\frac{1}{Q}\sum_{t=1}^{Q}x_{t}$$

where $x_{i}$ represents the ith attribute. Q represents the total number of samples.

Step 2 Calculate the covariance matrix C for all the attribute data, which can be represented by Equation (9).

(9) $$C=\frac{1}{Q}XX^{T}$$

where $X=\left\{x_{1},x_{2},\cdots ,x_{Q}\right\}$ represents the set of attributes affecting the disease diagnosis.

Step 3 Calculate the eigenvalues λ and eigenvectors μ of the medium covariance matrix C.

Step 4 Arrange the feature vectors into a matrix by the corresponding feature value size from top to bottom in rows. The cumulative information contribution corresponding to the eigenvalues can be expressed by Equation (10) and Equation (11).

(10) $$c_{l}=\frac{\lambda_{l}}{\sum_{u=1}^{Q}\lambda_{u}}\;l=1,2,\cdots ,Q$$

(11) $$\eta_{j}=\frac{\sum_{i=1}^{N}\lambda_{i}}{\sum_{i=1}^{Q}\lambda_{i}}\;j,N=1,2,\cdots ,Q$$

where $c_{l}$ represents the information contribution of the lth eigenvalue. $\eta_{j}$ represents the cumulative contribution of the former j principal components.

Step 5 Calculate the regression results. Calculate the loadings of each principal component on the variables. which can be represented by Equation (12).

(12) $$\begin{array}{l} s_{ij}=\iota \left(h_{i},g_{j}\right)=\sqrt{\lambda_{i}}\mu_{ij}\;i,j=1,2,\cdots ,Q\\ h_{i}=s_{i1}g_{1}+s_{i2}g_{2}+s_{i3}g_{3}+\cdots +s_{iQ}g_{Q} \end{array}$$

where $s_{ij}$ represents the variable load of $h_{i}$ to $g_{i}$. $\iota \left(h_{i},g_{j}\right)$ represents the calculation process for variable loads. $\mu_{ij}$ represents the jth component of the vector $\mu_{i}$. For the output score, a multiple linear fit using the selected Q principal components are performed to obtain the output regression model can be represented by Equation (13).

(13) $$score=r+\eta_{1}h_{1}+\eta_{2}h_{2}+\cdots +\eta_{Q}h_{Q}$$

where $\eta_{i}\left(i=1,2,\cdots ,Q\right)$ represents the weight coefficients of each principal component. r represents a constant.

### 3.3 The inference process of the model

The inference process of the H-BRBp is based on the evidential reasoning (ER) algorithm. This algorithm converts the belief degree to the underlying probability quality [30]. The specific inference steps are as follows:

Step 1: The output of the H-BRBp model needs to transform the input information first. The degree of matching between the input sample information and the belief rule is calculated. The calculation of the rule matching degree can be represented by Equation (14).

(14) $$a_{i}^{k}=\left\{\begin{array}{cc} \frac{A_{i}^{l+1}-x_{i}}{A_{i}^{l+1}-A_{i}^{l}} & k=l,A_{i}^{l}\leq x_{i}\leq A_{i}^{l+1}\\ 1-a_{i}^{k} & k=l+1\\ 0 & k=1,2,\cdots L,k\neq l,l+1 \end{array}\right\}$$

where $a_{i}^{k}$ represents the matching degree to the ith attribute. $x_{i}$ represents the value of the ith input attribute. $A_{i}^{l}$ and $A_{i}^{l+1}$ represent the reference values of two neighboring attributes.

Step 2: After the rule matching degree is obtained, the activation weight, i.e., the degree of activation of the input information on the rule, is calculated. The calculation of the activation weights can be represented by Equation (15).

(15) $$\omega_{k}=\frac{\theta_{k}\prod_{i=1}^{M}{\left(a_{i}^{k}\right)}^{\bar{\delta_{i}}}}{\sum_{l=1}^{L}\theta_{l}\prod_{i=1}^{M}{\left(a_{i}^{l}\right)}^{\bar{\delta_{i}}}},\bar{\delta_{i}}=\frac{\delta_{i}}{\begin{array}{c} \max\;\delta_{i}\\ i=1,2,\cdots ,M \end{array}}$$

where $\omega_{k}$ represents the activation weight. $\theta_{k}$ represents the rule weight. $a_{i}^{k}$ represents the matching degree. $\delta_{i}$ represents the attribute weight. $\bar{\delta_{i}}$ represents the relative attribute weights.

Step 3: Generate the belief degree of the model output using the ER parsing algorithm, which can be represented by Equation (16).

(16) $$\begin{array}{l} \beta_{n}=\frac{\mu \left[\prod_{k=1}^{L}\left(\omega_{k}\beta_{2^{N}}+1-\omega_{k}\sum_{j=1}^{2^{N}}\beta_{j}\right)-\prod_{k=1}^{L}\left(1-\omega_{k}\sum_{j=1}^{2^{N}}\beta_{j}\right)\right]}{1-\mu \left[\prod_{k=1}^{L}\left(1-\omega_{k}\right)\right]}\\ \mu =\frac{1}{\left[\sum_{n=1}^{2^{N}}\prod_{k=1}^{L}\left(\omega_{k}\beta_{2^{N}}+1-\omega_{k}\sum_{j=1}^{2^{N}}\beta_{j}\right)\right]-\left(2^{N}-1\right)\prod_{k=1}^{L}\left(1-\omega_{k}\sum_{j=1}^{2^{N}}\beta_{j}\right)} \end{array}$$

where $\beta_{i}$ represents the belief degree of the Nth output reference value.

Step 4: The output of the model can be represented by Equation (17).

(17) $$y_{r}=\sum_{n=1}^{2^{N}}z\left(D_{n}\right)\beta_{n}$$

where $y_{r}$ represents the actual output of the model. $z\left(D_{n}\right)$ represents the utility of $D_{n}$.

### 3.4 Optimization process of the H-BRBp disease diagnosis model

The parameters of the initial model are given by experts in the field with a combination of expertise and practical experience. The expert knowledge itself has some limitations. The mechanism of the disease diagnosis model is also very complicated. Therefore, it is difficult to determine accurate and reasonable parameter values for untrained models. Therefore, the parameters need to be fine-tuned by training the optimization algorithm to improve the accuracy of the model. The optimization objectives and constraints can be represented by Equation (18) and Equation (19).

(18) $$\begin{array}{l} \min\;MSE\left(\Omega \right)\\ s.t.\;\sum_{n=1}^{2^{N}}\beta_{n}=1\\ 0\leq \beta_{n}\leq 1,n=1,2,\cdots ,2^{N}\\ 0\leq \theta_{k}\leq 1,k=1,2,\cdots ,L\\ 0\leq \delta_{m}\leq 1,m=1,2,\cdots ,M \end{array}$$

(19) $$MSE\left(\Omega \right)=\frac{1}{NUM}\sum_{k=1}^{NUM}{\left(y_{r}-y_{p}\right)}^{2}$$

where MSE(Ω) represents the objective function of the optimization algorithm. $\Omega =\left\{\beta_{1},\beta_{2},\cdots ,\beta_{2^{N}},\theta_{1},\theta_{2},\cdots ,\theta_{L},\delta_{1},\delta_{2},\cdots ,\delta_{M}\right\}$. $y_{r}$ represents the evaluation results of the model. $y_{p}$ represents the true results in the sample. NUM represents the number of samples.

**Fig. 2:**
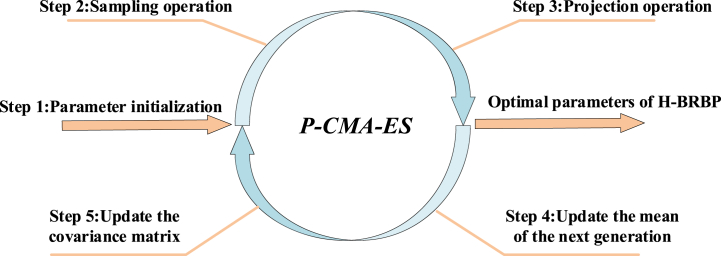
P-CMA-ES algorithm optimization process diagram.

Currently, many researchers have compared common optimization algorithms in BRBs. For example, Zhou et al. compared the constrained particle swarm algorithm (PSO), sequential quadratic programming (SQP), and projection covariance matrix adaptive evolution strategy (P-CMA-ES) in selecting a power set hidden belief rule base with power set (PHBRB) optimization algorithms. The experimental results showed that P-CMA-ES possesses better accuracy [31]. Cao et al. compared the optimization effectiveness of P-CMA-ES, the differential evolutionary algorithm (DE) and PSO and verified that P-CMA-ES has some interpretability while ensuring the optimization effectiveness [32]. As mentioned above, due to the superiority of P-CMA-ES. In this paper, P-CMA-ES is used as an optimization algorithm for the H-BRBp disease diagnosis model. P-CMA-ES is an improved algorithm in the original CMA-ES optimization algorithm for dealing with high-dimensional nonlinear optimization problems. The algorithm works by generating initial populations. Population selection is performed under the constraints, and the generation of subpopulations is continuously iterated to eventually find the optimal solution [33]. The steps of the P-CMA-ES optimization algorithm are shown in Fig. 2. The specific steps are as follows:

Step 1: Parameter initialization. Give the initialization parameters $w^{0}=\Omega^{0}$, which can be represented by Equation (20).

(20) $$\Omega^{0}=\left\{\beta_{1},\beta_{2},\cdots ,\beta_{2^{N}},\theta_{1},\theta_{2},\cdots ,\theta_{L},\delta_{1},\delta_{2},\cdots ,\delta_{M}\right\}$$

where $\Omega^{0}$ represents the set of parameters that need to be optimized.

Step 2: Generate offspring by a sampling operation, which can be represented by Equation (21).

(21) $$\Omega_{i}^{g+1}∼w^{g}+\varepsilon^{g}N\left(0,R^{g}\right),i=1,2,\cdots ,\lambda$$

where $\Omega_{i}^{g+1}$ represents the ith solution at the development to the (g+1)th generation. $w^{g}$ represents the mean value of the gth generation. $\varepsilon^{g}$ represents the step size of the gth generation. $R^{g}$ denotes the covariance matrix of the gth generation. N(·) represents the positive-terminus distribution. λ represents the number of offspring.

Step 3: Projection operation. Project the solution to the hyperplane. Constrain the parameters. This can be described as follows:

(22) $$\Omega_{i}^{g+1}\left(1+n_{e}\times \left(j-1\right):n_{e}\times j\right)=\Omega_{i}^{g+1}\left(1+n_{e}\times \left(j-1\right):n_{e}\times j\right)-\frac{F_{e}^{T}}{F_{e}\times F_{e}^{T}}\times \Omega_{i}^{g+1}\left(1+n_{e}\times \left(j-1\right):n_{e}\times j\right)F_{e}$$

(23) $$F_{e}\Omega_{i}^{g}\left(1+n_{e}\times \left(j-1\right):n_{e}\times j\right)=1$$

where Equation (22) is the constraint condition. Equation (23) is the hyperplane expression. $F_{e}$ represents the parameter vector. $n_{e}$ represents the constraint variables in $\Omega_{i}^{g}$. j represents the number of constraints in $\Omega_{i}^{g}$.

Step 4: Update the mean value of the next generation, which can be represented by Equation (24).

(24) $$w^{g+1}=\sum_{i=1}^{\sigma }h_{i}\Omega_{i:\lambda }^{g+1}$$

where $h_{i}$ represents the weighting factor. $\Omega_{i:\lambda }^{g+1}$ represents the ith solution in the (g+1)th generation λ solution. σ represents the progeny population size.

Step 5: Update the covariance matrix. Based on the initial covariance matrix and the optimal solution, the covariance matrix of the offspring population is updated so that the population approaches the optimal solution, which can be represented by Equation (25).

(25) $$R^{g+1}=\left(1-v_{1}-v_{2}\right)R^{g}+v_{1}P_{v}^{g+1}{\left(P_{v}^{g+1}\right)}^{T}+v_{2}\sum_{i=1}^{v}h_{i}\left(\frac{K_{i:\lambda }^{g+1}-\varphi^{g}}{\rho^{g}}\right){\left(\frac{K_{i:\lambda }^{g+1}-\varphi^{g}}{\rho^{g}}\right)}^{T}$$

where $v_{1}$ and $v_{2}$ represent the learning rate. $\rho^{g}$ represents the step size of the gth generation. $P_{v}^{g+1}$ represents the evolutionary path of the (g+1)th generation. $K_{i:\lambda }^{g+1}$ represents the ith parameter vector in the (g+1)th generation λ vector. $\varphi^{g}$ represents the progeny population of the gth generation.

Step 6 Repeat the above steps until the preset number or accuracy of training is reached.

### 3.5 H-BRBp disease diagnosis model development process

**Fig. 3:**
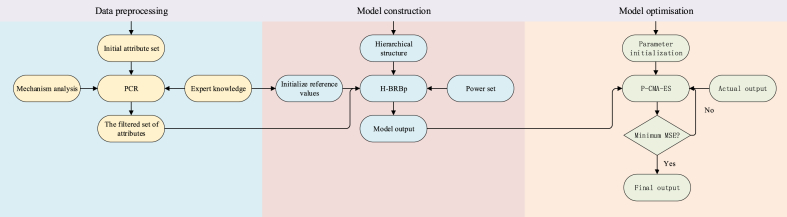
Process diagram of the H-BRBp disease diagnosis model.

According to Section 3.1 to Section 3.4, the overall flow chart of the H-BRBp disease diagnosis model is shown in Fig. 3. The specific process is described as follows:

Step 1: Data preprocessing.

Based on the data preprocessing process defined in Section 3.2. The input attributes of the H-BRBp disease diagnostic model are selected using the PCR algorithm based on Eqs. (9), (10), (11), (12), (13), (14) combined with mechanistic analysis of expert knowledge. The selected input attributes are divided into subsets, each of which corresponds to a set of inputs of the H-BRBp disease diagnosis model. This is shown in the data preprocessing section in Fig. 3.

Step 2: Model construction.

In the first step, the input set of the H-BRBp disease diagnosis model is obtained. And then the obtained input set is combined with the expert knowledge to construct the H-BRBp disease diagnosis model. The modeling process is based on the overall structure of the model defined in Section 3.1 and the model inference process defined in Section 3.3. The inference formulas defined by Eqs. (4), (5), (6), (7), (8), (9), (10), (11), (12), (13), (14), (15), (16), (17). The H-BRBp disease diagnosis model is constructed. The details are shown in the Model construction section in Fig. 3.

Step 3: Model optimization.

The output obtained in step 2 is the raw output of the H-BRBp disease diagnosis model. Its output is often less accurate. Therefore, the parameters of the H-BRBp disease diagnosis model can be optimized using Eqs. (18), (19), (20), (21), (22), (23), (24), (25) in the P-CMA-ES algorithm according to the optimization process defined in Section 3.3. This is shown in the model optimization part in Fig. 3.

## 4 Case study

In this section, an experimental sample of lumbar spine disease diagnosis is used to verify the validity and accuracy of the method proposed in this paper.

### 4.1 Data preprocessing

The dataset used in this paper is from the publicly available vertebral dataset in the UCI machine learning knowledge base. This dataset classifies patients into three categories, namely, healthy, herniated disc and lumbar spondylolisthesis. The sample sizes for the three types are 100, 60 and 150 cases, respectively. The data for each of these patient categories consist of six attributes: pelvic incidence (PI), pelvic tilt (PT), lumbar lordosis angle (LLA), sacral slope (SS), pelvic radius (PR), and grade of spondylolisthesis (GS). As described in Sections 2, 3 of this paper, if all the attributes were used as input attributes for BRBs in the experiment, the problem that would arise would be more than just a rule explosion problem. More importantly, when there are too many input attributes, the BRB model is easily disturbed by irrelevant attributes or attributes of lower importance, which leads to a reduction in the overall model accuracy. Therefore, to ensure the overall accuracy of the model, it is necessary to perform scientific selection of the attributes before the initialization of the model. In this paper, the PCR algorithm is used in combination with expert knowledge to select the input attributes of the lumbar spine disease diagnosis model species that have a high impact on the output results. The experimental results are shown in Table 1.

**Table 1:** Table of experimental results of the PCA algorithm.

Attribute	GS	LLA	PT	SS	PI	PR
Contributions	0.5406	0.7397	0.8667	0.9456	1	1

Remark: The value of contribution in the table represents the sum of the contribution of this attribute and the previous attributes to the result. For example, if the value of PT is 0.8667, it means that the set of attributes {GS, LLA, PT} contributes 0.8667 to the result.

According to the experimental results in Table 1, the contribution of the attribute set {GS, LLA, PT, SS} to the results can reach 0.9456. Although the contribution of the set of attributes {GS, LLA, PT, SS, PI} to the results can be as high as 1 if the attribute PI is also added to the set, the addition of the attribute PI only improves the results by 0.0544. This indicates that the contribution of the attribute PI to the results is negligible. Therefore, the experimental results show that determining the input set as {GS, LLA, PT, SS} can minimize the complexity of the model while guaranteeing its accuracy.

### 4.2 H-BRBp disease diagnosis model construction

**Fig. 4:**
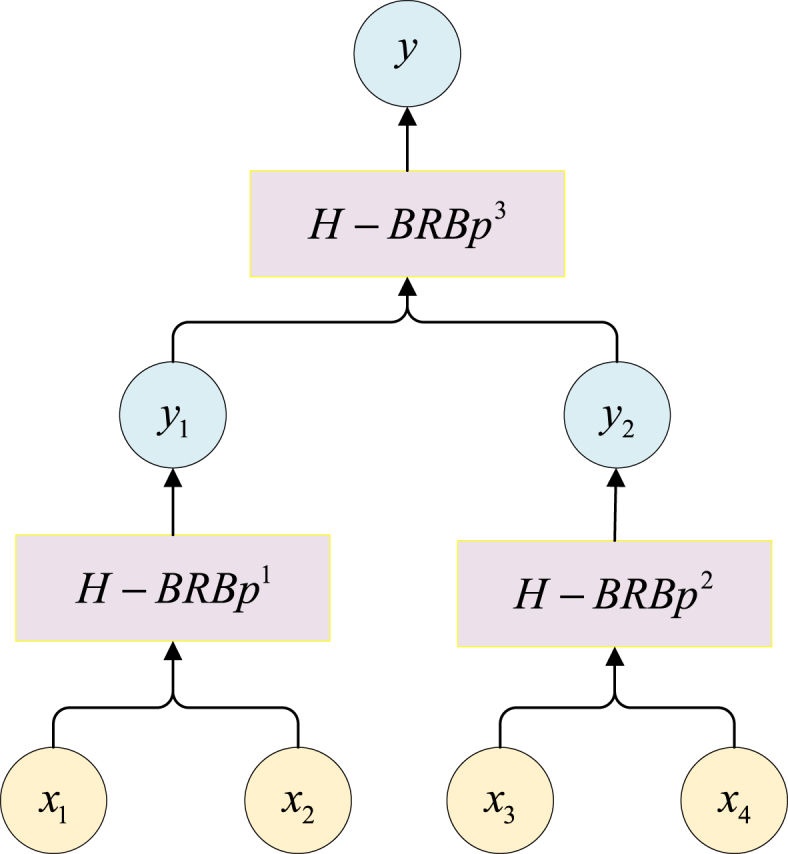
Process diagram of the experimental model.

Then, the input attribute set is obtained by the PCR algorithm. The H-BRBp disease diagnosis model can be divided into two layers and three sub rule bases, and the if-then rules for each layer of the H-BRBp model are shown in Equation (4) and Equation (8). There are two input attributes in each sub-rule base, and each input attribute has four reference values. Therefore, there are 16 rules in each sub rule base. The four input attributes in the model are $x_{1}$, $x_{2}$, $x_{3}$, and $x_{4}$, representing LLA, GS, PT, and SS, respectively. The basic flow of the experimental model is shown in Fig. 4. The reference values of each input attribute given by the expert knowledge are shown in Table 2. In the table, L represents low, M represents medium, H represents high, and VH represents very high. The reference values of the output results are shown in Table 3. In table G represents healthy, P represents disc-hernia, and O represents lumbar spondylolisthesis.

**Table 2:** Reference values for input attributes.

Reference Points				
Reference value	L	M	H	VH
$x_{1}$	13	35.9	49.3	126
$x_{2}$	−12	5	12.9	419
$x_{3}$	−7	15	25	50
$x_{4}$	13	33.4	41.99	122

**Table 3:** Reference values for output results.

Reference Points	G	P	O
Reference value	0	1	2

Remark: In this dataset, the input attributes of healthy and disc-hernia are highly similar, whereas the input attributes of lumbar spondylolisthesis are highly differentiated [34]. Therefore, in this experiment, there is local ignorance information between the two output outcomes of health and disc-hernia. In contrast, the global ignorance information and the empty set were almost negligible.

### 4.3 Experimental case analysis

Before proceeding to the case analysis, evaluation criteria need to be defined to measure the accuracy of the model. Two criteria are defined in this section, which are expressed as follows.

Criterion 1: Overall sample accuracy. The specific algorithm can be represented by Equation (26).

(26) $$All\_ac=\frac{correct}{all}\times 100$$

where correct represents the number of samples with correct diagnosis results. all represents the number of all samples tested.

Criterion 2: Accuracy of disease samples. The specific algorithm can be represented by Equation (27).

(27) $$Illness\_ac=\frac{IN^{\prime }}{IN}\times 100$$

where IN represents the total number of disease samples. $IN^{\prime }$ represents the number of samples that can be correctly diagnosed with the disease type.

The results obtained after the experimental steps and parameter settings defined in Section 4.2 are unoptimized results. Due to the limitations of expert knowledge, the results obtained are often not very accurate. To solve this problem, the H-BRBp disease diagnosis model is optimized using the P-CMA-ES algorithm based on the optimization model in Section 3. In this section, $H-BRBp^{1}$ is used as an example, and its optimized parameters are shown in Table 4.

**Table 4:** $H-BRBp^{1}$ Optimized attribute parameters.

Rule	Rule weights	Attribute		Distribution of output
		$x_{1}$	$x_{2}$	{G, {G, P}, P, {P, O}, O}
1	0.0918	L	L	{0.9648,0.0281,0.0063,0.0002,0.0005}
2	0.0005	L	M	{0.0015,0.3633,0.4511,0.1685,0.0156}
3	0.0003	L	H	{0.1712,0.4618,0.0418,0.0014,0.3238}
4	0.9916	L	VH	{0.6518,0.1531,0.1921,0.0006,0.0024}
5	0.001	M	L	{0.005,0.0265,0.2919,0.4669,0.2097}
6	0.4231	M	M	{0.9283,0,0.0704,0.0013,0}
7	0.2775	M	H	{0.996,0.0028,0.0004,0,0.0008}
8	0.0084	M	VH	{0.379,0.2716,0.3,0.0279,0.0215}
9	0.4142	H	L	{0.3503,0.1475,0.426,0.0111,0.065}
10	0.1211	H	M	{0,0,0,0.0948,0.9052}
11	0.4192	H	H	{0.0001,0.0006,0.0025,0.0319,0.9649}
12	0.0013	H	VH	{0.0981,0.0734,0.4412,0.3775,0.0098}
13	1	VH	L	{0.1889,0.4101,0.0033,0.0928,0.3049}
14	0.0211	VH	M	{0.0741,0.0007,0.1537,0.3269,0.4446}
15	0.0477	VH	H	{0.7073,0.0031,0.2879,0.0004,0.0013}
16	0.0006	VH	VH	{0.1990,0.4714,0.0027,0.1807,0.1462}

**Fig. 5:**
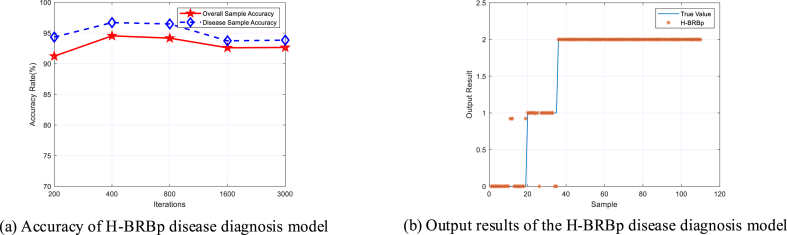
H-BRBp disease diagnosis model.

The accuracy of the model in the H-BRB disease diagnosis model is related to the number of iterations. The number of iterations is also proportional to the running time of the system. During the experiment, the number of iterations of the model was set to 200, 400, 800, 1600, and 3000. Two hundred cases are used as training samples, and 110 cases are used as test samples (in the subsequent comparison experiments). The training and test sets will be randomly sampled and used to verify the effectiveness of the model in the presence of small random samples. The accuracy of the experimental results for different iterations is shown in Fig. 5 (a). Fig. 5 (a) shows that the accuracy of the model is highest when the number of iterations is 400, and the small number of iterations ensures a short running time of the system. The output of the H-BRBp disease diagnosis model after determining the number of iterations is shown in Fig. 5 (b). From Fig. 5 (b), it can be seen that the output of the H-BRBp disease diagnosis model has a good fit with the actual values.

**Fig. 6:**
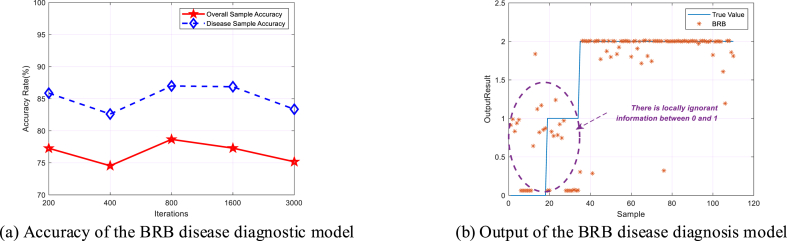
BRB disease diagnosis model.

**Fig. 7:**
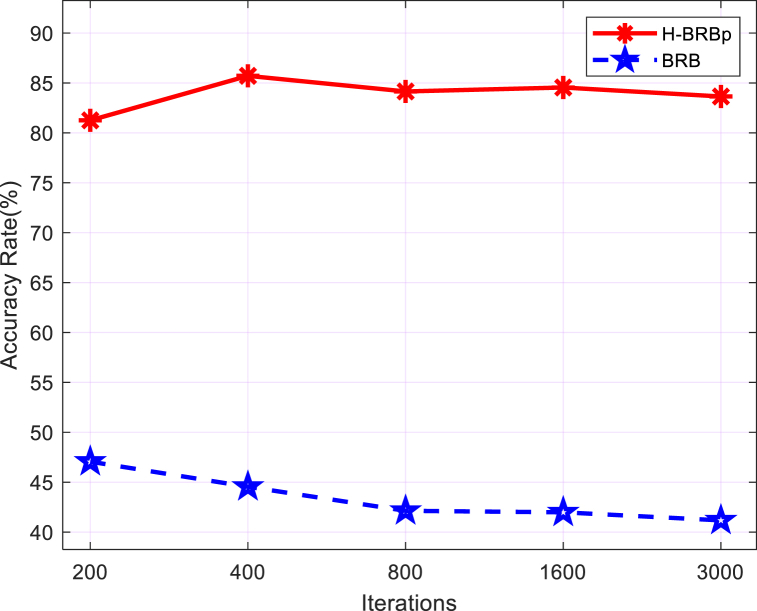
Accuracy of the H-BRBp model compared with the BRB model in the 0–1 interval.

To verify that the H-BRBp disease diagnosis model can effectively handle local ignorance information. The BRB was applied to this experiment. The same optimization algorithm was used for the experiments. Its overall sample accuracy and disease sample accuracy are shown in Fig. 6 (a). The fit of its output results to the actual values is shown in Fig. 6 (b). The experimental results show that the BRB disease diagnosis model can obtain a high accuracy rate when 800 iterations are performed, but only 78.6598%. This is much lower than the 94.5455% accuracy of the H-BRBp disease diagnosis model at 400 iterations, and the time required to run the model is also longer. More importantly, the purple circle in Fig. 6 (b). The BRB disease diagnosis model can be seen in the purple circles in the healthy group represented by 0 and the disc herniation interval represented by 1, where the predicted results differ from the true data by a large result. This is because in reality, the healthy lumbar spine and the disc hernia have similar properties. This leads to the existence of local ignorance information between 0 and 1. The BRB model cannot handle the local ignorance information well. However, the H-BRBp can handle this local ignorance information well. The accuracy of the H-BRBp model and BRB model in the interval of 0–1 is shown in Fig. 7. The accuracy of the H-BRBp model in dealing with local ignorance information is almost twice that of the BRB model. Therefore, the experimental results show that the H-BRBp model not only has a higher accuracy rate compared with the BRB but can also handle local ignorance information very well.

### 4.4 Comparison test

**Fig. 8:**
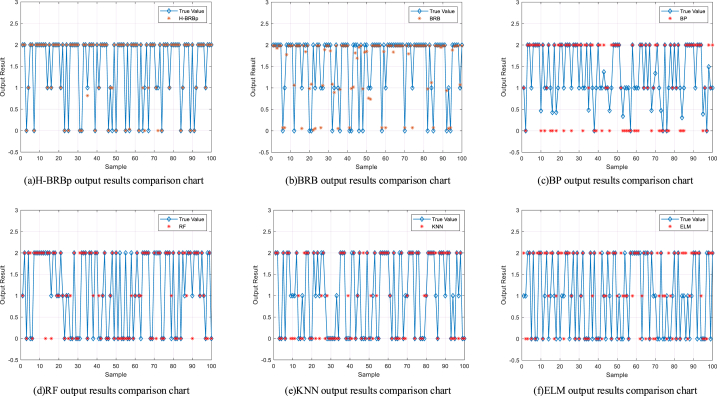
Comparison of the output results of different models with the real loss results.

In this section, H-BRBp, BRB, BP Neural Network (BP), Random Forest (RF), K-Nearest Neighbor (KNN), and Extreme Learning Machine (ELM) are used to perform comparative experiments for the diagnosis of lumbar spine diseases. The same training set and test set were used for all methods. Twenty rounds of experiments were repeated, and in each round, 100 samples were randomly selected from the training set to train the model. In the test set, 100 samples were randomly selected to verify the output effect. The evaluation criteria are shown in Equation (26). One round is randomly selected among 20 rounds of experiments to compare the output of different methods with the real output. The experimental results are shown in Fig. 8.

**Fig. 9:**
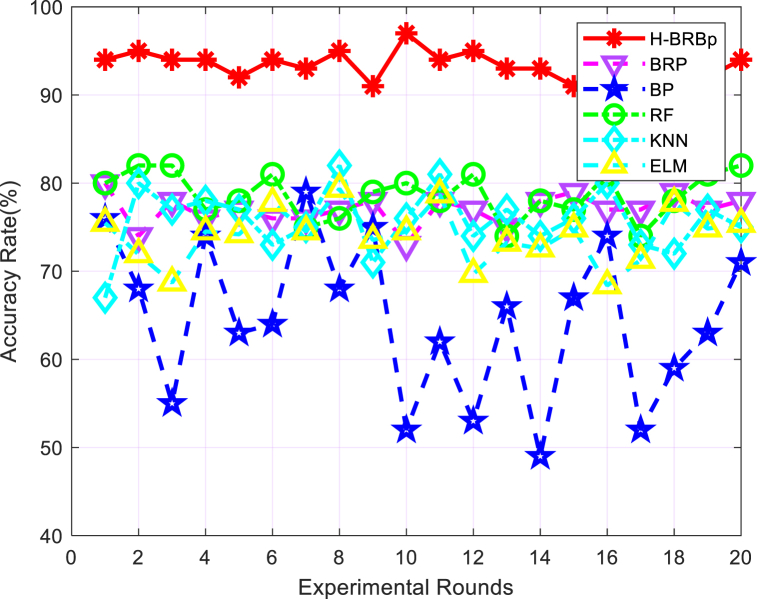
Comparison chart of the accuracy rates of different algorithms.

The accuracy comparison graph of different methods for the diagnosis of lumbar spine diseases is shown in Fig. 9. The average accuracy of different algorithms after 20 rounds of experiments is shown in Table 5. Fig. 9 and Table 5 show that compared with other methods, the H-BRBp disease diagnosis model has the highest accuracy rate. After many rounds of experiments, the H-BRBp disease diagnosis model has good stability, and its accuracy rate can be maintained above 90% all the time. The accuracies of the BRB, RF, KNN, ELM and other algorithms are basically between 70% and 80%. Although BRBs have a lower accuracy rate, their stability is good. This is related to the interpretability of the BRB model itself. The worst performer is the BP algorithm. It is not only that its accuracy is the lowest. More importantly, its model is less stable and has less credibility in the context of practical medical applications.

**Table 5:** Average accuracy of 20 rounds of experiments with different models.

Model	H-BRBp	BRB	BP	RF	KNN	ELM
Accuracy	93.65%	77%	64.5%	78.7%	75.75%	74.05%

In the construction of various disease diagnostic models, analysis of their model complexity is necessary. In this paper, we analyze the complexity of training and complexity of space for six disease diagnostic models in our experiments. Training complexity can be viewed as a measure of how quickly a machine learning algorithm can be executed for the input size. Spatial complexity can be viewed as the amount of additional memory required to execute the machine learning algorithm. The results of the analysis are shown in Table 6 where P−CMA−ES represents the training complexity of this disease diagnosis model and is dependent on its optimization algorithm. rule represents the number of rules. n represents the number of training samples. d represents the data dimension. k represents the number of neighbors. s represents the number of decision trees [[35], [36], [37], [38]].

**Table 6:** Comparison table of the complexity of different disease diagnostic models.

Model	Complexity of training	Complexity of space
H-BRBp	P−CMA−ES	O(rule)
BRB	P−CMA−ES	O(rule)
BP	O(n*d)	O(d)
RF	O(n*log(n)*d*s)	O(p*s)
KNN	O(k*n*d)	O(n*d)
ELM	O(n*d)	O(d)

Through experiments, it was found that the reasons for the lower accuracy and lower stability of the remaining methods were as follows:

1) The H-BRBp model is a transparent inference mechanism. Its rules are built based on expert knowledge with interpretability. However, the internal structure of BP, RF, KNN, ELM and other models is not visible. The inference process of the model lacks interpretability and cannot explain the relationship between input and output.
2) The H-BRBp model is less sample dependent. When random sampling is performed, the sample type is likely to be incomplete. The rest of the models are less able to handle the situation when facing incomplete sample types.
3) Compared with the H-BRBp model, the remaining models cannot handle the local ignorance information well. For example, the input attributes between output type 0 and output type 1 in this sample have a strong similarity, which easily leads to local ignorance of information between 0 and 1.

To conclude, through experiments, we found that H-BRBp disease diagnosis has good ability. Not only has a high accuracy rate, but the model also has a high stability and the ability to deal with local ignorance information. Finally, H-BRBp disease diagnosis is less sample dependent. It also has good performance when the training sample type is not complete.

### 4.5 Ablation research

In this paper, the experimental sessions can be divided into three parts: data preprocessing, model training and comparison tests.

1. Data preprocessing

Data preprocessing ensures the accuracy, completeness, consistency, credibility, and interpretation of experimental data. The PCR algorithm was used in this experiment to process the lumbar spine disease data from the UCI machine learning knowledge base. The interference attributes and attributes with low contribution values in the dataset were screened out, which contributed to the accuracy of the H-BRBp disease diagnosis model and largely reduced the complexity of the model. Therefore, the data preprocessing part in this paper plays a role in laying the foundation of the overall experiment. If this part is removed, the construction of the H-BRBp disease diagnosis model will become extremely complicated.

2. Model training

When evaluating a disease diagnosis model, its accuracy is the primary consideration. While the quality of the algorithm of a disease diagnosis model can affect the accuracy to some extent, a more important factor is whether the training set of the model is well chosen. Especially when there is an imbalance in the proportion of disease types in the training sample, whether the disease diagnosis model has sufficient adaptability is also the key to measuring the model. In this paper, the training set selection in the comparison experiments was determined by a random sampling method. The accuracy of the H-BRBp disease diagnostic model did not vary greatly during the 20 rounds of random sampling. Therefore, the adaptation of the H-BRBp disease diagnosis model can be judged visually by this experimental session. Therefore, the model training part in this paper is the core part of the H-BRBp disease diagnosis model in the experiment. If this part is removed, the accuracy of the H-BRBp disease diagnosis model will not be guaranteed.

3. Comparison tests

The comparison experiment is the closing part of the experimental session. In this paper, the accuracy of the experimental results was analyzed by constructing equation (26), and the ability of the H-BRBp disease diagnosis model to handle local ignorance information was analyzed by constructing equation (27). Finally, the advantages of the model were verified by comparing it with the BP, RF, BRB, KNN, and ELM models through 20 rounds of random sampling experiments. Therefore, the comparison experimental part of this paper is an important part used to verify the advantages and disadvantages of the disease diagnosis models. If this section is removed, the validity and advantages of the models will not be verified.

## 5 Conclusion

In this paper, an H-BRBp disease diagnosis model was designed. The input attributes of the model were first selected using the PCR algorithm. Then, the H-BRBp model is constructed based on expert knowledge. The rule explosion problem brought by the excessive number of input attributes and the local ignorance problem brought by the high similarity of input attributes are successfully solved. Finally, a lumbar spine disease diagnosis experiment was performed to verify the performance of the model. The experimental results showed that the accuracy of the H-BRBp disease diagnosis model reached more than 90%. The accuracy of processing local ignorance information can also reach more than 80%. In contrast, the accuracy of BRB for local ignorance information is only approximately 40%. At the end of the experimental section, the superiority and stability of the model are further verified by comparison experiments with BRB, BP, KNN, RF, ELM and other models. Due to its expert knowledge background, the model itself has a certain degree of interpretability. It is also important to note that H-BRBp can be applied to data models with labels, which can be either quantitative information or qualitative knowledge. However, for the field of graphic image processing, H-BRBp has some limitations. For processing information such as graphical images, feature extraction of data samples is required in advance to transform them into labeled data samples. In future research, we will carry out the following research:

1) New disease diagnosis data need to be extracted and used to further train the model to improve its accuracy again.
2) During the optimization of the H-BRBp disease diagnosis model, further research on its interpretability can be considered.

## Author contribution statement

Wenzhe Han: Conceived and designed the experiments; Performed the experiments; Analyzed and interpreted the data; Wrote the paper.

Kang Xiao; He Wei: Conceived and designed the experiments; Analyzed and interpreted the data; Wrote the paper.

Li Jiang: Conceived and designed the experiments; Analyzed and interpreted the data; Contributed reagents, materials, analysis tools or data.

Hongyu Li; Bing Xu: Contributed reagents, materials, analysis tools or data.

## Funding statement

Wei He was supported by Postdoctoral Science Foundation of China under Grant [2020M683736], Teaching reform project of higher education in Heilongjiang Province under Grant [SJGY20210456 and SJGY20210457], Natural Science Foundation of Heilongjiang Province of China under Grant [LH2021F038], haiyan foundation of Harbin Medical University Cancer Hospital under Grant [JJMS2021-28], graduate academic innovation project of Harbin Normal University under Grant [HSDSSCX2022-17, HSDSSCX2022-18 and HSDSSCX2022-19].

## Data availability statement

Data associated with this study has been deposited at http://archive.ics.uci.edu/ml

## Declaration of interest’s statement

The authors declare no competing interests.

## Additional information

No additional information is available for this paper.
